# *Viva la* VOSCE?

**DOI:** 10.1186/s12909-020-02444-3

**Published:** 2020-12-18

**Authors:** J. G. Boyle, I. Colquhoun, Z. Noonan, S. McDowall, M. R. Walters, JP. Leach

**Affiliations:** 1 Department of Medicine, Glasgow Royal Infirmary, Castle Street, NHS GGC, Glasgow, G4 0SF UK; 2 School of Medicine, Dentistry and Nursing, University of Glasgow, Glasgow, G12 8QQ UK

**Keywords:** COVID-19, Assessment, OSCE

## Abstract

**Background:**

The COVID-19 pandemic lockdown precluded face-to-face final Objective Structured Clinical Examinations (OSCE) in the UK.

**Results:**

In response, we rapidly developed and then successfully implemented a novel Virtual Objective Structured Clinical Examination (VOSCE).

**Conclusions:**

In this article we both describe and reflect on our experience as well as discuss the implications for future undergraduate assessment as the situation evolves.

## Main text

The COVID-19 pandemic lockdown in the UK precluded our planned face-to-face final Objective Structured Clinical Examinations (OSCE). We required a high-stakes summative clinical examination to demonstrate competence among medical students who had raised concerns in previous clinical attachments. In response, we conceived, developed and then successfully implemented a novel Virtual Objective Structured Clinical Examination (VOSCE) using videotelephony through a cloud-based peer-to-peer software platform (Zoom™). Experienced OSCE examiners were recruited and given online training on the new format and software functionality. Students received an online briefing, and individual advance testing was performed to ensure that their homes had the appropriate bandwidth, and that their hardware had application compatibility. Students were instructed to complete the examination alone with other applications closed for the duration of the examination. The time between inception to delivery of the VOSCE was around 2 weeks.

Our OSCE blueprint, constructs, instructions and structured marking schedules were adapted to recognise that, in the absence of simulated or real patients, we would not be able to directly observe physical examination or communication skills. Stations were based on a short case vignette. As a proxy for history taking skill assessment, students were questioned about features from the patient’s history they would ask about to formulate their diagnosis in each case. The technical capability to share clinical photographs, laboratory results and imaging sequentially using a screen-sharing facility enabled the assessment of data interpretation and clinical reasoning domains as usual via examiner questioning. Structured questions and pre-defined checklists were carefully developed to examine learning objectives that were high across both the cognitive process (higher order thinking skills) and knowledge dimensions (abstract knowledge) [1]. Examples of such learning objectives include ‘Differentiate’ (Analyse and Conceptual knowledge) and ‘Judge’ (Evaluate and Procedural knowledge). We made adaptations, cognizant of the need to maintain content validity and careful blueprinting continued to challenge the vexing issue of context specificity. The use of multiple stations and examiners was retained from the OSCE format for reliability purposes. During the VOSCE, examiners rotated around rooms without any difficulty as the student interface remained on the same remote workstation. This allowed each examiner to assess the same station to maintain internal consistency.

On reflection, we learned that our cohesive team was able to mobilise quickly and work dynamically, interdependently and adaptively to the time-sensitive nature of this task. This required effective teamwork and successful engagement in this novel taskwork. We believe that communication, co-ordination and collaboration proved to be key to the smooth execution of our inaugural VOSCE; the circuits ran to time, with examiners maintaining a socially distanced ‘chain’ around stations. This included clear communication with students about the aims and process as well as the co-ordination of feasibility checks to ensure that we had the necessary technical capability. The rapid enforced adaptation to online education meant that faculty were already familiar with the functionality of the application. Team collaboration and communication facilitated the development of shared mental models of the task at hand, thereby minimising any costs of time and short-term working memory during the delivery of the VOSCE.

There was little doubt that the inability to involve real or simulated patients interfered with the authenticity of each stations’ stimulus and our revised VOSCE format represented a ‘best-case’ scenario. While we were unable to assess the ‘whole task’ as defined by our OSCE blueprint, the VOSCE did afford the opportunity to assess many components of clinical reasoning (including hypothesis generation, problem representation, differential diagnosis, leading diagnosis, diagnostic justification and management) [2]. The information gathering process is a critical component of clinical reasoning, and one of the advantages of the OSCE over other summative assessments. A potential limitation of this format is the need to infer the information gathering process from the open loop narrative generated when students were probed about additional features of the history in the absence of the facility for patient feedback. The use of video-clips and interactive simulation of patients (ISP) was considered but precluded due to the agility the rapid adaptation required. We believe the VOSCE format also has potential as a flexible formative assessment tool, especially if combined and triangulated with other recordings of history, examination or communication skills.

As with any cloud-based software platform security issues such as data privacy and unwanted intrusion (Zoom-bombing) remain a concern. Remote-proctoring software was not available for use in our institution at the time of delivery. We would argue that the near-constant interaction with examiners would make collusion or extraneous help almost impossible (and the need for additional invigilators present unnecessary). Fortunately, we did not have any technical issues but in such high stakes examinations this remains a tangible risk. Stations were therefore recorded with the students’ permission in case we were subject to adverse circumstances that were beyond the student’s control and affected performance. All students were provided with written feedback on station performance and fail students were provided with further individualised feedback via Zoom™.

Despite the lack of simulated or real patient contact, the VOSCE was both feasible to deliver and acceptable to students (no concerns or complaints received during or after the examination diet) and examiners (personal communications); enabling a summative assessment of clinical performance in a virtual format [3]. While it is not without its’ challenges, we plan to return to the traditional gold standard OSCE format when the COVID-19 situation allows, acknowledging that the unprecedented events of recent weeks strengthen the case for implementing elements of programmatic assessment into our undergraduate curriculum [4, 5].

## Data Availability

N/A
